# Meetings of Societies

**Published:** 1897-03

**Authors:** 


### MEETINGS OF SOCIETIES.

### Bristol Medico-Chirurgical Society.

*December 9th*, 1896.

Dr. A. E. Aust Lawrence, President, in the Chair.

Dr. J. Lacy Firth showed a specimen of multiple aneurysms. There was one of each axillary artery and of the innominate artery and of the right carotid. The specimen had been obtained from a man aged 68. He had bronchitis of twenty years' duration, and surgical interference did not seem called for. The signs of intrathoracic aneurysm were pulsation in the second right intercostal space and impaired percussion resonance of the pulsating area.

Dr. James Swain showed a renal calculus which, three months ago, he had removed from a patient of Mr. T. F. Edgeworth's. The diagnosis had been made clear by skiagraphy (see p. 9).—Dr. Theodore Fisher mentioned that he had seen a renal calculus removed by Mr. Jacobson which was covered over with small shining crystals of oxalate of lime.

Mr. Arthur W. Prichard showed two patients whom he had trephined for compound depressed fracture of the frontal bone (see p. 27).—Mr. J. Ewens mentioned the case of a child admitted into the Children's Hospital with an abscess over the left parietal eminence. The child had a stone thrown at her three weeks previously. She was stunned, but soon rallied, and excepting some vomiting and headache for three or four hours, suffered no inconvenience, and ran about as usual, swelling and abscess following. On opening the abscess, pus and brain-substance exuded through an opening in the skull of the size of a florin. The skin was replaced and the wound healed kindly. No brain symptoms followed. The child remained in the hospital for a month, and was discharged cured. When seen some years after, there had been extensive reproduction of bone; only a very small soft spot to be felt in the centre.

Dr. F. H. Edgeworth narrated a case of poisoning by tomatoes. A boy was found lying unconscious in the street, with a half-emptied box of tomatoes beside him, and was brought to the Bristol Royal Infirmary on the evening of July 16th. He subsequently confessed that he had been steadily eating them all day long. On admission he was unconscious and collapsed, but after a few hours partially recovered his senses. During many hours he repeatedly vomited and passed half-ripe decomposing tomatoes. The temperature gradually rose, and for the next week varied between 99° and 101.5°, then sank to normal and remained so. The semi-conscious condition persisted for three days after admission, and was succeeded by one of mental weakness and irritability lasting for nearly a fortnight. In addition, however, there was much intolerance of light, together with severe frontal headache and retraction of the head; and on July 21st, five days after admission, there was double optic neuritis fairly well marked.

These signs of meningitis gradually subsided and disappeared within four weeks. The muscles of the limbs wasted, with loss of knee-jerks and slight electrical degenerative reaction in the extensor muscles of knees and anterior tibial muscles; but there was no anæsthesia of the skin nor tenderness of the nerves and muscles, nor were there any pains radiating down the nerves. The peripheral neuritis, evidenced by these signs, was never very acute and slowly subsided; and the boy went home well, six weeks after admission. This case affords some evidence that both meningitis and peripheral neuritis may result from food-poisons, and suggests that in cases where the etiology of such conditions is doubtful, enquiry should be made for previous symptoms, such as gastro-intestinal irritation, which might give a clue towards the discovery of such a case.—Dr. T. Fisher referred to the point of interest of the case upon which Dr. Edgeworth laid stress—namely, that the food-poisoning occurred through eating vegetable, but not decomposing animal matter. He mentioned that a certain number of deaths from food-poisoning were recorded annually during the months of July, August, and September, and that probably others occurred which were not recorded. He had made two *post-mortem* examinations during the months of July and August, 1896, in which no cause for death could be found, which he attributed to food-poisoning. In the previous year he had made a *post-mortem* examination on a similar case.—Dr. H. Waldo said Dr. Edgeworth's case reminded him of a patient he took into the Bristol Royal Infirmary some years ago with gastro-enteritis and collapse. The patient, a young girl, had partaken of tinned salmon; she presented signs of peripheral neuritis, her knee-jerks were absent, and there was double optic neuritis present. At the time Dr. Waldo attributed the symptoms to ptomaine poisoning.—Mr. Paul Bush asked if anyone else partook of the fruit, or if it was ascertained whether the fruit came from abroad, as in that case it might easily be decomposed.—Dr. G. Parker mentioned two cases of probable food-poisoning occurring soon after each other in the same house. The first commenced with violent vertigo, which continued some days. There was also vomiting, a very rapid heart, albuminuria, and persistent localised pain in the right side of the abdomen. After a slow recovery of his patient, a married daughter had a similar attack, where the abdominal pain persisted some weeks and was accompanied with pain and tenderness in the limbs. After these symptoms had passed away, marked wrist-drop and difficulty in standing followed.—Dr. Shingleton Smith alluded to the botanical aspects of the tomato, the Lycopersicum esculentum of the East Indies, and its association with the other Solanaceæ, most of which are poisonous, but one (the Solanum tuberosum) a common article of diet. He thought that in this case the quantity of tomatoes eaten was a more important point than the presence of anything positively poisonous.—Dr. A. Carr mentioned a case which came under his notice in which a boy of ten years, of weak intellect, was found very faint and ill with vomiting and diarrhœa. He had eaten freely of potato apples. He was cured by a mustard emetic.—Dr. J. E. Shaw said that the tomato, upon its introduction from America towards the end of the 16th century, was known as the "Love-apple," and was supposed to be possessed of aphrodisiac properties. It is figured and so described in Gerarde's *Herbal*, published in 1597.—Dr. Markham Skerritt thought that possibly decomposition taking place in the alimentary canal itself might aid in producing toxic symptoms.

*January 13th, 1897.*

Dr. A. E. Aust Lawrence, President, in the Chair.

Dr. H. Waldo showed a man, aged 42, a labourer by trade, who was admitted to the Bristol Royal Infirmary on November 11th, 1896. His symptoms, together with the physical signs, suggested a deep-seated saccular aneurysm of the second part of the aortic arch. The diagnosis seemed confirmed by the tracheal tugging which was a well-marked feature of the case.—Mr. W. J. Penny cited the case of a man, aged about 46, who died suddenly with copious hemorrhage from the mouth. The necropsy showed a fusiform aneurysm of the arch of the aorta, which was atheromatous and calcified. Admitting a finger-tip, there was an ulceration through into the right bronchus. There had been no symptoms pointing to aneurysm, and the man had followed his work as a plumber.

Dr. Theodore Fisher showed several mounted pathological specimens recently added to the Bristol Royal Infirmary Museum. A few, such as the cavity of a subphrenic abscess, hernia of the bladder, rupture of aneurysm into the right ventricle, and cystic glioma of the brain, were shown for their intrinsic interest. Others, such as hemorrhage into a phthisical cavity, hemorrhage into the brain, and a diffuse aneurysm of the axilla, were shown to illustrate the way in which specimens can be mounted so as to retain their colour.

Dr. W. H. C. Newnham reported three cases of vaginal hysterectomy for cancer (see p. 24).—Dr. W. C. Swayne said that the important points appear to be the questions of diagnosis and fitness for operation. The question of the relative merits of partial or total extirpation requires time for its decision. On *à priori* grounds the radical operation is superior to partial removal, in that it is a more correct surgical procedure. As regards the diagnosis, in any case of hemorrhage after the menopause, and in all cases of menorrhagia and metrorrhagia, careful investigation for the purpose of excluding cancer is necessary. Fœtid discharge resembling that of cancer may be caused by senile endometritis, but a careful curetting will clear up any doubt that may appear to exist. Pain is a doubtful sign, and many cases far too advanced for removal are met with in which pain has not been marked. How far the question of infiltration should contraindicate removal is an important point. The opinion of those who have operated on many cases would be valuable as to how far fixation and apparent infiltration may be caused by inflammatory deposit due to septic absorption. —Dr. J. G. Swayne agreed with Dr. Newnham that cancer in such young subjects was doubtless due to rapid child-bearing in girls in their teens, owing to its inducing premature old age and exhaustion. He thought that prophylactic treatment at an early period was the most effectual remedy, *i.e.* to prohibit marriage before twenty, especially in girls of delicate constitution.—Dr. Michell Clarke, with regard to the early age at which carcinoma appeared in Dr. Newnham's patients, referred to the views of Ribbert and others, who trace the origin of cancerous growths to separation of epithelial cells from their normal connections, the separated cells either perishing, remaining dormant, or taking on growth according to the variations in the surrounding conditions, and suggested that rapidly repeated pregnancies would afford the necessary conditions for the separation and abnormal growth of epithelial cells.—Mr. Greig Smith, referring to Dr. Michell Clarke's remarks on the origin of cancer, said that although the resting bud theory would account for the hypertrophic element in cancerous growths, it would not account for the cachexia. He dwelt upon the importance of early diagnosis, particularly in cases originally in the body of the uterus. The hypertrophic adenoid material found in certain cases not cancerous might also be found in cases which have cancer at the bottom, and there may be infiltrating new growth in the uterine body and little or no discoverable lesion in the mucosa. As to the question of total or partial removal, he was on the whole inclining to the complete operation; but he was not prepared definitely to recommend it for all cases. In the most skilled hands a mortality of from five to eight per cent. was all that was incurred, and it was doubtful if the lower mortality of the partial operation would counterbalance this.—Mr. C. A. MORTON referred to a case in which he performed the operation last summer. The woman was excessively anæmic from uterine hemorrhage, and he gave ʒiss. of calcium chloride during the twenty-four hours preceding the operation. Although the operation was a very difficult one, owing to extension of the disease to the vaginal wall around the cervix and to marked elongation of the body of the uterus, there was no great amount of bleeding, and the patient made a satisfactory recovery. Mr. Morton had also employed this method of trying to diminish the tendency to hemorrhage during an operation in other very anæmic patients, and with apparent success. He did not wish to discuss the question of the relative merits of complete hysterectomy or supra-vaginal amputation, but he would like to refer to the difficulty in deciding, during the performance of the operation, whether the disease was limited to the cervix or had extended into the body of the uterus. If, after separation of the bladder and ligation of the base of the broad ligaments, the cervix was grasped between the thumb and finger, it was impossible to define the limits of the growth, as the uterine tissue was as hard or harder than the cancerous growth. The best plan, he considered, was to perform a supra-vaginal amputation of the cervix—and he was himself in favour of this operation rather than complete hysterectomy in suitable cases, —and then, if on examination of the removed cervix the disease was found infiltrating up to the line of section or very near it, to proceed to complete removal of the uterus. This was the plan which he intended to adopt in a case of carcinoma of the cervix upon which he was just going to operate.—Mr. J. EWENS called attention to a paper read by Dr. Macpherson Lawrie, of Weymouth, at the Carlisle meeting of the British Medical Association, reporting six consecutive cases of successful vaginal hysterectomy.—The PRESIDENT drew attention to the great importance of attention to all irregularities of menstrual function in women who have not passed the climacteric, and to the occurrence of vaginal bleeding in those who have passed the climacteric He advocated the total removal of the uterus rather than partial.—Dr. NEWNHAM, in reply, said that having been present at the meeting of the British Gynæcological Society when Jessett read his paper on seventy-five cases of vaginal hysterectomy for malignant disease, he was much struck by the fact that all the speakers were agreed that there was no doubt about the justifiability of the operation, and the ease and success with which it could be done; but each one asked for the early symptoms of malignant disease which could lead to a diagnosis. The ordinary signs, such as pain, fœtid discharge, and hemorrhage, generally only appear when the disease is too far advanced for operation. He then suggested that the small hemorrhage occurring after coitus is often a sign, which should always be viewed with great suspicion, and imperatively demands a vaginal examination.

Dr. B. M. H. Rogers described the case of a girl, aged 12, who he thought suffered from mediastinitis. She had been quite well till the summer of 1896, when œdema of the legs and ascites had been noticed. When she came under his care at the Children's Hospital the œdema extended as high as the clavicles, and there was considerable ascites and the veins of the neck were distended. There was no displacement of the heart, no murmur to be detected except a *bruit de galop*, no physical signs of any disease of the chest or presence of a new growth in the mediastinum, and the urine was normal. During her stay in the hospital the symptoms slightly improved, and after four months she went to Weston. On her return her condition was not so satisfactory. The heart was displaced upwards and to the left, the œdema and ascites had increased, and she was much weaker. Dr. Rogers discussed the diagnosis between tubercular disease of the glands, lymphadenoma, acute inflammation, and new growths, and concluded that the case was one of chronic inflammation of the posterior mediastinum.—Dr. T. Fisher considered that those writers who believe mediastinitis to be capable of producing symptoms overlook one or two anatomical facts. The inferior vena cava has too short a course in the chest to be pressed upon by contraction of fibrous tissue, and it is the superior vena cava only that is worth considering in connection with this point. The superior vena cava will, however, bear great compression, without signs of venous obstruction becoming manifest. The great cause, and it might be said the only cause, of such obstruction is produced by growth, and that does so by invading the interior of the vessel and obliterating its lumen. Mediastinitis might lead to venous obstruction by producing thrombosis, but this is exceedingly rare. Dr. Fisher acknowledged thickening of the tissues of the mediastinum to be common in cases of adherent pericardium, but distension of the veins, when present, is due to the cardiac failure.

---

*February 10th*, 1897.

Dr. A. E. Aust Lawrence, President, in the Chair.

Dr. F. H. Edgeworth showed a patient, aged 52 years, suffering probably from an intrathoracic tumour. He had been losing weight, had cough and expectoration, for eight months. There was no displacement of the intrathoracic organs, nor evidence of any pressure on intrathoracic veins, arteries or nerves. The respiratory murmur was everywhere deficient. There was some deficiency of resonance over the manubrium sterni. The glands in the anterior triangle of the neck and axilla were hard and enlarged on both sides, though more marked on the right side. The glands in the groins were not enlarged. The interest of the case lay in the slightness of the intrathoracic signs, though the glands in the neck and axillæ were considerably enlarged.—Dr. J. O. Symes said that in view of the fact that glands at such distant points as the groin and over mastoid process were affected, it was unlikely that their enlargement was due to secondary infection from a growth in the thorax.—Dr. J. Broom suggested that skiagraphy would probably remove some obscure parts of the present diagnosis.

Dr. T. Fisher showed a further collection of pathological specimens from the Infirmary museum. As before, the series contained some specimens, such as tuberculosis of the lung, broncho-pneumonia, and fibroid disease of the heart, mounted in order to preserve their colour; and others of more special interest, such as ulcerative endocarditis, showing healing of the valves, primary growth of the base of the lung, and softening of the brain following thrombosis of the superior longitudinal sinus.

Mr. C. W. J. Brasher showed a modification of Hale White's well-known tongue-depressor for infectious cases, both instruments consisting essentially of a Turck's tongue-depressor carrying a glazed "window" by means of an attached arm; Dr. Hale White's instrument having the arm fixed to the depressor by two small screws, requiring a screwdriver to detach them. The modified instrument has the arm attached by means of a clamp, allowing of ready disconnection of the arm for disinfection, &c. The attachment of the "window" to the arm in the modified instrument has a winged nut in addition to a thumb-screw, and by this means a double movement of the "window" is permitted. Any obscuration by the patient's breath may be prevented by warming the glass before use.

Dr. Michell Clarke related the case of a man who, after inhaling sewer gas, suffered from fœtor of breath, paroxysmal cough with abundant very fœtid expectoration, heavy sweats, hectic fever and emaciation. Two months from the beginning of the illness the physical signs pointed to the presence of an abscess in the lower lobe of the right lung, and Mr. C. A. Morton by an operation which he described reached a small abscess cavity in the midst of consolidated lung in this situation. The abscess was drained and the man was doing well, when he had a series of epileptic fits—from which he had suffered for some years—and died in coma.—Dr. C. Elliott said that all such cases when hectic symptoms set in invariably died if treated only by medicine. The rule he would lay down was this, that as soon as there was unmistakable fœtor there should be no delay, but surgical assistance should be sought for, even though the abscess cavity might be small, as necrotic changes were taking place rapidly. Hitherto most surgeons had been unwilling to operate in these cases.—Mr. J. Ewens mentioned a case in which he himself had aspirated an abscess of the lung in a child in the Children's Hospital, with good results, the diagnosis of abscess of lung being materially aided by the fœtid nature of the expectoration.—Dr. G. Parker mentioned the early symptoms of the case while it was under his care, and the difficulty of diagnosis before the fœtor became noticeable.—Dr. T. Fisher referred to a case in the Bristol General Hospital which he had seen while taking Dr. Michell Clarke's work, in which the diagnosis of the abscess cavity was easy and the situation favourable for operation; but the condition was thought too acute, with too much accompanying septic pneumonia, for surgical interference, and in a few days perforation occurred into the pleural cavity.—Dr. A. J. Harrison mentioned the case of an oldish man admitted under his care in the Bristol General Hospital a good many years ago, before the discovery of Koch's bacillus, with symptoms of breakdown of left apex of lung. Signs local; breath fœtid; probable cause, apical pneumonia previously. Mr. Penny, the house surgeon, tapped the apex and drained with tube. The man made a perfect recovery.—Dr. Markham Skerritt spoke of the difficulty which had repeatedly been met with in accurately localising by physical signs the position of a cavity in the lung, and referred to a case recorded by Sidney Coupland in which all the evidence of a large cavity existed, but none could be reached by operation; *post-mortem* examination proved that a congeries of dilated bronchial tubes had given rise to the physical signs characteristic of a single cavity.—Mr. Morton referred to a case very similar to Dr. Coupland's which he had published in the *Lancet*, 1894.

Dr. Barclay J. Baron read a paper on "The Symptoms and Treatment of Chronic Suppuration of the Maxillary Antrum," and Mr. W. H. Harsant commented on the paper (see pp. 13—20).

Dr. G. Parker read a paper on the diagnosis of certain affections allied to rheumatism. He referred to the difficulty of distinguishing the rheumatic group of affections from others in the absence of any exact criterion of rheumatism, and argued that in certain diseases we have definite symptoms and sequelæ sufficient to differentiate them from rheumatism, and to enable us to place under these diseases many ill-defined cases which are often regarded as rheumatism. Such diseases are Quincke's œdema, Osler-Henoch's purpura (which often has no skin affections at all), rheumatoid arthritis, the arthritis of gonorrhœa, scarlatina, diphtheria, and other exanthemata, purpura hemorrhagica, hæmophilia, malignant endocarditis, &c. Many of these are absolutely distinct from rheumatism, and the evidence for the rheumatic nature of even erythema nodosum is most defective. Cases were given in illustration of these propositions, showing nervous, renal and pyrexial symptoms unknown in rheumatism. The danger of relying on a history of previous rheumatic attacks which might refer to very different diseases, or on the evidence of endocarditis produced by various causes, was also discussed.—Dr. A. J. Harrison emphatically objected to erythema nodosum being classed with rheumatism. Erythema nodosum may occur in rheumatic subjects, but there is much about the complaint which at present is very obscure. Thus it is *rare* for a person to have it twice, although rheumatism is very recurrent. One attack of erythema nodosum seems to exhaust its incidence, or when it does recur a person may have it two or three times, just as a very few people may have small-pox, measles, or scarlet fever several times, but single attacks are by all means the rule. We have a good deal to learn about this form of erythema.

J. Paul Bush, *Hon. Sec.*

### Devon and Exeter Medico-Chirurgical Society.

*Annual Meeting, January 15th*, 1897.

Mr. J. D. Harris, President, in the Chair.

The Council's Report and the Treasurer's Report having been received, the following officers for the ensuing year were elected:—President; Dr. J. Somer: Vice-President; Dr. A. G. Blomfield: Treasurer; Mr. J. D. Harris: Council; Dr. R. Thomas, Mr. A. C. Roper, Dr. H. Davy, Dr. F. Morgan, Dr. J. K. Lewis, and Dr. C. N. Lovely: Auditors; Dr. W. Budd and Dr. J. Mackeith: Hon. Secretary; Dr. R. V. Solly.

Dr. Ransom Pickard read a paper on "Syphilitic Joint-disease." He also reported a case of deafness due to congenital syphilis where, after *post-nasal* adenoids had been removed without benefiting the hearing, interstitial keratitis and syphilitic joint-disease developed, and then almost complete recovery of hearing was obtained under mercurial treatment.

Dr. Mark Farrant, jun., showed a case of deafness due to congenital syphilis, with interstitial keratitis, in which no improvement took place under mercurial inunction.

R. V. Solly, *Hon. Sec.*

### Plymouth Medical Society.

*October 3rd.* 1896.

Dr. C. A. Hingston, President, in the Chair.

Dr. J. Webber read notes oi a case of diphtheria and shewed a specimen of membrane.

Mr. R. H. Lucy showed specimens of sarcoma of lower jaw, calcareous bronchial glands, and ingrowing toe-nail.

Mr. C. E. Russel Rendle handed round a card specimen of a middle meningeal artery ruptured in two places.

Mr. A. Rendle showed a young woman whose apex-beat was to be felt on the right side.

---

*October 21st*, 1896.

Dr. C. A. Hingston, President, in the Chair.

Mr. Lucy read a paper on the "Surgical Treatment of Hemorrhoids."

---

*November 7th*, 1896.

Dr. C. A. Hingston, President, in the Chair.

Mr. W. L. Woollcombe showed: (1) A woman from whose neck he had removed a sarcomatous mass, including ¾ of clavicle, large portion of internal jugular and subclavian veins, and an inch of the thoracic duct. After three months the patient did not appear much deteriorated. (2) A man with left popliteal aneurysm, apparently fusiform. (3) A calculus, 1¾-in. long and 186 grs. in weight, removed *per urethram* from a child, aged 3½ years, with no permanent damage to urethra.

Mr. C. E. Russel Rendle demonstrated a case of capsular cataract with extreme myopia and choroidal atrophy.

Mr. G. F. Aldous exhibited a skiagram of compound fracture of radius and ulna, and handed round portions of former bone removed for cure of resulting deformity.

Mr. W. L. Woollcombe showed a specimen of appendix vermiformis removed during herniotomy for obstructed inguinal hernia, in whose sac was found the fundus of cæcum, part of a sacculus of the bladder, and much condensed fat.

Mr. R. H. Lucy showed a small case of instruments for intra-venous injection of saline solution.

Dr. Pethybridge exhibited a piece of catheter removed from membranous portion of urethra by Dr. Webber.

---

*Annual Meeting, November 18th*, 1896.

Dr. C. A. Hingston, President, in the Chair.

Mr. J. H. S. May was elected President for the ensuing year, and Mr. W. L. Woollcombe, Hon. Sec. The other office-bearers remain the same.

The members dined together at the Duke of Cornwall Hotel.

*November 28th*, 1896.

Mr. G. JACKSON in the Chair.

Mr. RIDER showed a child with congenital morbus cordis and an inversion of the skin over the sacro-coccygeal junction.

Mr. WOOLLCOMBE showed: (1) A woman with secondary syphilis and nodes on cranium and right clavicle. (2) A child, aged 3½, with congenital loss of voice, due to an arrest of development of the larynx. (3) A child, aged 15 months, with various developmental defects, including an absence of lower epiphysis of right tibia but normal growth of fibula, so that upper end of fibula articulated with external condyle of femur and lower end projected beyond foot, causing extreme inversion.

Mr. RIDER read notes of a case of rapidly fatal peritonitis, supposed to be the result of ruptured appendix, with specimen.

Dr. J. WEBBER showed a microscopic slide of rodent ulcer.

Mr. C. H. WHITEFORD read notes of a case of apical pneumonia.

---

*December 16th*, 1896.

Mr. J. H. S. MAY, President, in the Chair.

Dr. W. C. WILSON read a paper on "The Treatment of Insomnia."

---

*January 9th*, 1897.

Mr. J. H. S. MAY, President, in the Chair.

Dr. E. LAURENCE FOX showed a man, aged 20, with functional spasm in one arm and hand.

Mr. C. E. RUSSEL RENDLE showed a case of fracture through epiphysis of metacarpal of thumb in a lad aged 19, illustrated by a skiagram of both hands.

Mr. C. H. WHITEFORD read (1) notes of a case of misplaced testis in a child aged 2, the organ lying in the perineum; (2) notes of a case of deafness resulting from long-standing otorrhœa, hearing being reduced to a watch at three inches. Under $CHCl_3$ both membranes were found to be destroyed, and a large crop of adenoids was removed from the naso-pharynx as a preventive of future complications. This had the effect of completely restoring the hearing.

Dr. J. WEBBER showed a slide of blood-clot from a case of extra-uterine gestation, operated on by Mr. W. L. Woollcombe in tenth week; the specimen showed well the presence of chorionic villi.

---

*January 27th*, 1897.

Dr. E. LAURENCE FOX in the Chair.

Dr. J. WEBBER read a paper on "The Present State of Serum-therapeutics," the diseases taken to illustrate the paper being diphtheria, tetanus, inoperable malignant disease, and inflammations caused by streptococcus pyogenes.

*Card Specimen.*—Mr. C. H. Whiteford: The bladder and right kidney and the shrivelled remains of left ureter, which opened into left seminal vesicle and was filled with a thin, opaque, light-brown fluid and a nodule of hardened fat which was all that represented the left kidney. The bladder was much thickened and sacculated, and the right kidney was in the state known as "surgical kidney." The man, aged 68, died suddenly of uræmia.

W. L. WOOLLCOMBE, *Hon. Sec.*

